# Experimental validation of absolute SPECT/CT quantification for response monitoring in patients with coronary artery disease

**DOI:** 10.1186/s40658-021-00393-4

**Published:** 2021-06-16

**Authors:** Alina van de Burgt, Petra Dibbets-Schneider, Cornelis H. Slump, Arthur J. H. A. Scholte, Douwe E. Atsma, Lioe-Fee de Geus-Oei, Floris H. P. van Velden

**Affiliations:** 1 Department of Radiology, Section of Nuclear Medicine, Leiden University Medical Center, PO Box 9600, 2300 RC Leiden, The Netherlands; 2 Technical Medical Center, University of Twente, Enschede, The Netherlands; 3 Department of Cardiology, Leiden University Medical Center, Leiden, The Netherlands; 4 Biomedical Photonic Imaging Group, University of Twente, Enschede, The Netherlands

**Keywords:** ^99m^Tc-tetrofosmin, SPECT/CT, Experimental validation, Quantitative SPECT, Coronary artery disease, Phantom study

## Abstract

**Background:**

Quantitative SPECT enables absolute quantification of uptake in perfusion defects. The aim of this experimental study is to assess quantitative accuracy and precision of a novel iterative reconstruction technique (Evolution; GE Healthcare) for the potential application of response monitoring using ^99m^Tc-tetrofosmin SPECT/CT in patients with coronary artery disease (CAD).

**Methods:**

Acquisitions of an anthropomorphic torso phantom with cardiac insert containing defects (with varying sizes), filled with ^99m^Tc-pertechnetate, were performed on a SPECT/CT (Discovery 670 Pro, GE Healthcare). Subsequently, volumes of interest of the defects were manually drawn on CT to assess the recovery coefficient (RC). Bull’s eye plots were composed to evaluate the uptake per segment. Finally, ^99m^Tc-tetrofosmin SPECT/CT scans of 10 CAD patients were used to illustrate clinical application.

**Results:**

The phantom study indicated that Evolution showed convergence after 7 iterations and 10 subsets. The average repeatability deviation of all configurations was 2.91% and 3.15% (%SD mean) for filtered (Butterworth) and unfiltered data, respectively. The accuracy after post-filtering was lower compared to the unfiltered data with a mean (SD) RC of 0.63 (0.05) and 0.70 (0.07), respectively (*p* < 0.05). More artificial defects were found on Bull’s eye plots created with the unfiltered data compared to filtered data. Eight out of ten patients showed significant changes in uptake before and after treatment (*p* < 0.05).

**Conclusion:**

Quantification of ^99m^Tc-tetrofosmin SPECT/CT seems feasible for CAD patients when 7 iterations (10 subsets), Butterworth post-filtering (cut off frequency 0.52 in cycles/cm, order of 5) and manual CT-delineation are applied. However, future prospective patient studies are required for clinical application.

**Supplementary Information:**

The online version contains supplementary material available at 10.1186/s40658-021-00393-4.

## Introduction

Myocardial perfusion imaging is used to evaluate the presence and severity of coronary artery disease (CAD) [1, 2]. Myocardial perfusion scintigraphy (MPS) using single photon emission computed tomography (SPECT), typically obtained on a CZT camera, is the most extensively validated imaging modality for this purpose [3] and is routinely used to manage treatment strategies [2, 4]. Moreover, monitoring the response of CAD treatments with MPS may guide treatment decision making.

MPS is based on visual interpretation of relative myocardial perfusion and might underestimate the severity of ischemia due to global hypoperfusion [5]. Hence, it is worth investigating how quantitative SPECT may enable measurement of uptake in perfusion defects to improve evaluation of response to anti-ischemic therapies using myocardial perfusion scans.

Recent developments in iterative imaging reconstruction, such as Evolution (Q.Metrix package, GE Healthcare, Milwaukee, USA) available on a Xeleris workstation (version 4.0) [6], allows SPECT to provide absolute quantification. Evolution is an ordered subset expectation maximization algorithm that includes compensation for collimator–detector response, attenuation and scatter correction, and resolution recovery. Evolution has been validated for oncological trials using phantom studies for assessing early response to treatment in locally advanced breast cancer patients [7] and for the differentiation of normal bone and bone disease [8]. Nonetheless, to the best of our knowledge, no validation has been performed for cardiac studies.

Therefore, the aim of this experimental study is to assess quantitative accuracy and precision of Evolution, making use of phantom studies, supplemented with illustrative patient cases, for potential clinical application of response monitoring using ^99m^Tc-tetrofosmin SPECT/CT in patients with CAD.

## Materials and methodology

### Phantom studies

**Fig. 1:**
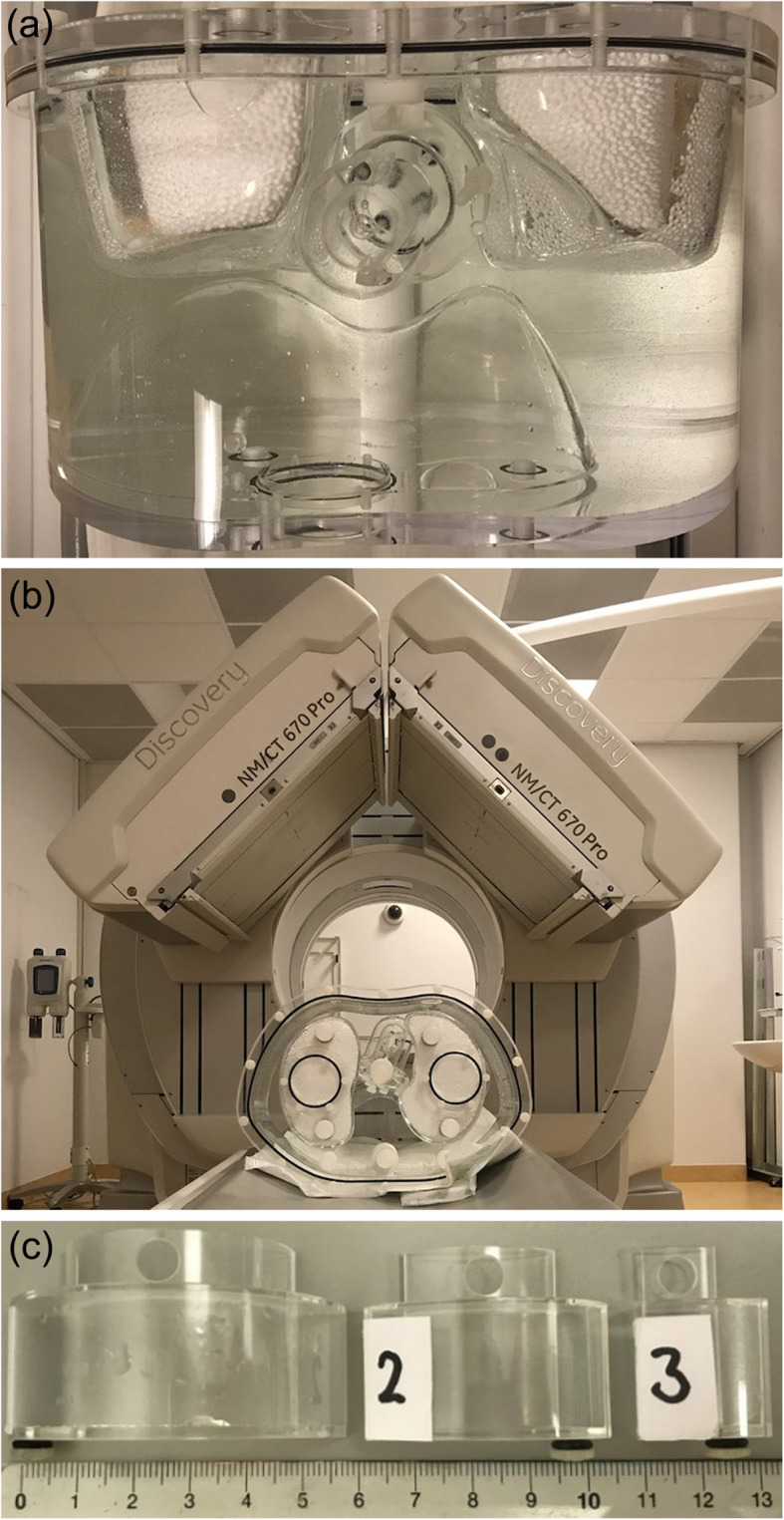
Axial view of an anthropomorphic torso phantom containing a cardiac insert, spine, lungs and liver (**a**). Positioning of the phantom on the SPECT/CT scanner (**b**). Three defects with various sizes and scale in cm (**c**)

In an anthropomorphic torso phantom (model ECT/TOR/P, DATA Spectrum, Hillsborough, NC, USA) a static cardiac insert was used (Data spectrum cardiac phantom model ECT/CAR/UM, DATA Spectrum, Hillsborough, NC, USA; Fig. 1), consisting of a left ventricle with separate compartments for blood pool (with a volume of 61 mL) and myocardium (with wall thickness of 10 mm and volume of 100 mL). The insert compartments were filled with a solution of water and ^99m^Tc-pertechnetate. Three fillable defects (small, 2.6 mL; medium, 5.6 mL; and large, 11.8 mL) were evaluated to simulate a myocardial perfusion defect. The defects were positioned in the anterior or inferior wall of the myocardium, or a combination of both. The cardiac insert was positioned in an anthropomorphic torso phantom containing lung, liver and spine inserts. The myocardium was filled with 64 kBq/mL. Moreover, the liver was filled with 15.9 kBq/mL according to a myocardium-to-liver ratio of 4:1. The myocardium-to-background ratio was approximately 12:1. In addition, the myocardium-to-blood pool ratio was 10:1 and the defect-to-myocardium ratio was 1:2, based on literature [9–11]. The lungs were filled with polystyrene spheres and water to achieve a representative physiologic tissue density.

A total of 36 measurements were acquired and evaluated: six configurations with six acquisitions. The first three single configurations are the described fillable defects measured separately on the mid anterior part of the myocardium, further referred to as small (S-configuration), medium (M-configuration) and large (L-configuration). The fourth and fifth configurations are two combinations of two defects and were positioned on the mid-inferior and mid-anterior part of the myocardium. These double defect configurations consist of two small defects (SS-configuration) and a combination of a small and medium defect (SM-configuration). Finally, the phantom was also scanned without defects, further referred to as the ground truth (GT-configuration). The measurements were repeated six times with a time per view that was adjusted per repetition to compensate for the radioactive decay and to have similar count statistics as the first experiment.

Data acquisition and reconstruction were based on the clinical protocol using one bed position according to the EANM guidelines [12]. All studies were performed with a SPECT/CT dual head system (Discovery NM/CT 670 Pro, GE Healthcare, Milwaukee, Wisconsin, USA). The SPECT measurements are acquired using a low-energy, high-resolution (LEHR) collimator that was positioned in L mode, noncircular orbit, step-and shoot mode and 60 (2 × 30) views. The technetium energy window (photopeak) was set on 140.5 keV (window ±10%) for emission and on 120 keV (window ±5%) for scatter. The camera sensitivity was determined as recommended by the vendor [6] as described in more detail in Collarino et al. [7]. After the SPECT acquisition, a low-dose CT scan (120 kV, 20 mAs, pitch 0.938, collimation 16 × 1.25) was acquired for attenuation correction purposes.

All SPECT data were reconstructed using Evolution with compensation for collimator-detector response, resolution recovery, attenuation and scatter on a matrix of 64 × 64 voxels with 1.5 zoom, resulting in a voxel size of 5.89 × 5.89 × 5.89 mm^3^. Moreover, the CT data were reconstructed using adaptive statistical iterative reconstruction (ASIR, GE healthcare) with a voxel size of 0.98 × 0.98 × 5.00 mm^3^.

Based on the recommendations of the vendor, no post-reconstruction filter should be applied for quantification [6]. However, a Butterworth filter (cut-off frequency of 0.52 cycles/cm and an order of 5) was recommended by the vendor for clinical SPECT/CT cardiac studies without quantification. Therefore, SPECT data were reconstructed both with and without a Butterworth filter to investigate the impact of post-filtering on quantification. After reconstruction, the Q.Metrix package resampled both the CT and SPECT images to an equivalent and isotropic voxel size (1.47 × 1.47 × 1.47 mm^3^) that was used for delineation.

#### Data analysis

All SPECT/CT images were converted from counts to Bq/mL using Q.Metrix as detailed in the paper of Collarino et al. [7]. To determine the number of iterations that are required for Evolution to converge, volumes of interest (VOI) were drawn on CT images for the background, liver, small defect and myocardium compartments. Evolution was considered to have converged when for each VOI the relative difference in the activity concentration of the iteration was less than 1% with respect to the previous iteration. In order to determine the noise level, the coefficient of variation (COV) in the background compartment was calculated by the standard deviation divided by the mean activity concentration.

Interobserver reliability of delineation on CT images was assessed by three physicians that draw VOIs on the small defect and the myocardium for the S-configuration. The interobserver reliability was computed by an intraclass correlation coefficient using a two-way mixed effects model for absolute agreement (ICC_A_) (SPSS statistics; version 25; IBM Statistics, Armonk, USA). ICC scores range from 0 to 1, representing a level of agreement: ≤ 0.40 poor to fair, 0.41–0.60 moderate, 0.61–0.80 substantial and 0.81–1.00 almost perfect [13].

Subsequently, recovery coefficients (RCs), representing the ratio between the reconstructed activity concentration (in Bq/mL) and the true activity concentration as measured with a dose calibrator (VIK-202, Comecer, the Netherlands), were computed for VOIs drawn on CT images for the myocardium and defect compartments for all configurations. Here, an RC of 1 indicates that injected activity concentration was in accordance with the measured activity concentration. Furthermore, for all six configurations separately, the precision was expressed in average repeatability deviation (RD) calculated by the standard deviation (SD) of the myocardium RCs as a percentage of the mean myocardium RC. Moreover, Bull’s eye plots with 17 segments were generated using 4DM (INVIA-Ann Arbor, MI, USA) for the S- and GT-configurations.

### Patient cases

Patient cases were evaluated retrospectively to illustrate the clinical applicability of the phantom study. For patients with CAD and ischemia without long-term treatment options, intramyocardial injection of autologous bone marrow cells (BMC) has emerged as an alternative treatment strategy at Leiden University Medical Center (LUMC) [14]. SPECT/CT data were composed before and after BMC injection. The collected patient data were anonymized and recorded in a database. Performance of this retrospective study was approved by the medical ethical review board and the requirement to obtain informed consent was waived.

Comparing the MPS at rest and after stress, areas of under-perfusion and resultant stress-induced ischemia were identified. According to a two-day protocol, as described in the EANM guidelines, patients under and over 100 kg received 500 and 750 MBq ^99m^Tc-tetrofosmin, respectively, for both the stress and the rest examination [12]. All patients underwent a pharmacologic adenosine stress examination. SPECT/CT was acquired 30–45 min post-injection after stress and 45–60 min post-injection during rest.

All stress and rest studies were converted into lean-body mass standardized uptake values (SUV_LBM_) in g/mL and calculated as described in Kim et al. [15]. Reversibility Bull’s eye plots were composed for the studies before and after BMC injection as used in clinical practice [12]. Subsequently, the difference in tracer uptake (SUV_LBM_) prior to and after BMC injection was calculated by the reversibility Bull’s eye plots after BMC injection minus before BMC injection.

### Statistical analysis

Statistical analysis was performed using SPSS statistics software and Excel (version 2017; Microsoft, Redmond, USA). A Shapiro-Wilk test was performed in order to evaluate the (log)normality of the data. Paired data for both the phantom and the patient study were statistically performed with the paired T test or Wilcoxon Signed Rank test depending on the (log)normality. The statistical analyses of the non-paired data were performed with an independent T test or Mann-Whitney U depending on the (log)normality. Continuous data were expressed as mean(SD).

## Results

### Phantom study

#### Camera sensitivity, convergence and noise level

**Fig. 2:**
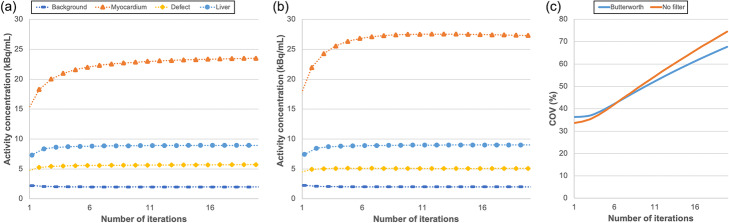
Effects of increasing the number of iterations for Evolution on the reconstructed activity concentrations of background, myocardium, small defect and liver compartments, obtained with (**a**) and without (**b**) Butterworth post-filtering, and on (**c**) COV derived from the background compartment

The camera sensitivity was 74.0 cps/s/MBq. Evolution showed convergence for all investigated VOIs when at least 7 iterations (and 10 subsets) were applied (Fig. 2). Moreover, an increasing noise level (COV) was shown with increasing number of iterations. With 7 or more iterations, the filtered data showed a lower noise level compared to the unfiltered data. At 7 iterations, the COV was 44.3% and 44.5%, with and without Butterworth post-filtering, respectively. For the remainder of the study, all data were reconstructed using 7 iterations and 10 subsets.

#### Interobserver variability

Almost perfect agreement between the observers was obtained when the small defect and myocardium were delineated manually on CT images (ICC_A_ ≥ 0.863 and ≥ 0.839, respectively).

#### Recovery coefficients for myocardium compartment

RC for the filtered data was significantly lower compared to the unfiltered data (*p* < 0.05, Table 1). All mean RCs were significantly smaller (up to 0.3 in mean RC) for the filtered data compared to the unfiltered data. However, SD values were similar with and without filtering in all configurations. No significant difference was shown between filtered RD and unfiltered RD (*p* > 0.05). The largest RD (5.53 and 5.41 with and without Butterworth filtering, respectively) was depicted for the GT configuration and the smallest RD (1.18 and 1.53 with and without Butterworth filtering, respectively) for the configuration with the two small defects.

**Table 1:** Mean recovery coefficient (RC), SD and repeatability (RD), obtained with and without Butterworth post-filtering, derived from the myocardium compartment for various configurations

	No filter			Butterworth		
Configuration	*RC*	*SD*	*RD*	*RC*	*SD*	*RD*
S	*0.67*	*0.02*	*3.51*	*0.64*	*0.02*	*2.72*
M	*0.63*	*0.01*	*1.59*	*0.61*	*0.01*	*1.41*
L	*0.62*	*0.03*	*4.91*	*0.59*	*0.03*	*4.35*
SS	*0.60*	*0.01*	*1.53*	*0.58*	*0.01*	*1.18*
SM	*0.63*	*0.01*	*1.96*	*0.61*	*0.01*	*2.26*
GT	*0.63*	*0.03*	*5.41*	*0.61*	*0.03*	*5.53*
Mean			***3.15***			***2.91***

#### Recovery coefficients for defects

**Fig. 3:**
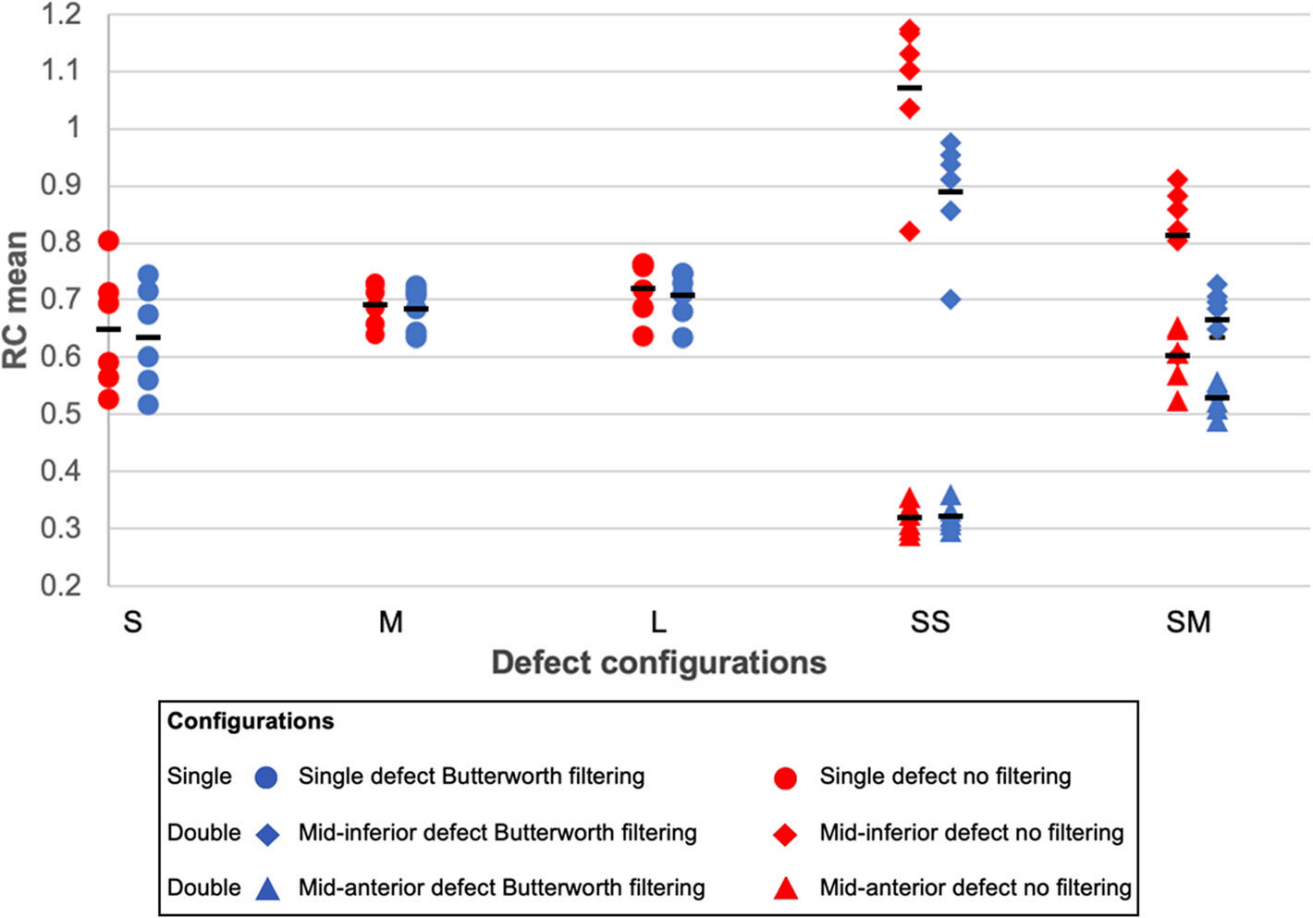
Recovery coefficients (RC) of single and two defects: small (S), 2.6 mL; medium (M), 5.6 mL; and large (L), 11.8 mL. The defect RC of Evolution with (blue) and without Butterworth post-filtering (red). Moreover, RC mean of the defect was illustrated separately for the SS and SM-configurations

Filtered data showed a significantly lower mean (SD) RC over all defect volumes compared to the unfiltered data with 0.63 (0.05) and 0.70 (0.07), respectively (*p* < 0.05; Fig. 3). The variability between the acquisitions with the same configuration was, however, significantly (*p* < 0.05) larger in the unfiltered data (maximum RD difference of 2.74% between filtered and unfiltered data). In addition, various similar patterns were seen in both graphs. First, the mean RC values in smaller defect volumes were lower compared to larger defect volumes with 0.65 and 0.72 for the unfiltered data and 0.64 and 0.71 for the filtered data, respectively (*p* < 0.05). Second, smaller defect volumes yielded larger variations between the repetition measurements with maximum RD values of 12.4% and 10.4% for unfiltered and filtered data, respectively. Third, the apical defects showed significantly (*p* < 0.05) lower RC values compared to the basal defects, with RC values of 0.32 and 0.60 for the unfiltered data and 0.32 and 0.53 for the filtered data. Finally, a large variation between the two small defects was found.

#### Bull’s eye plots variability

**Fig. 4:**
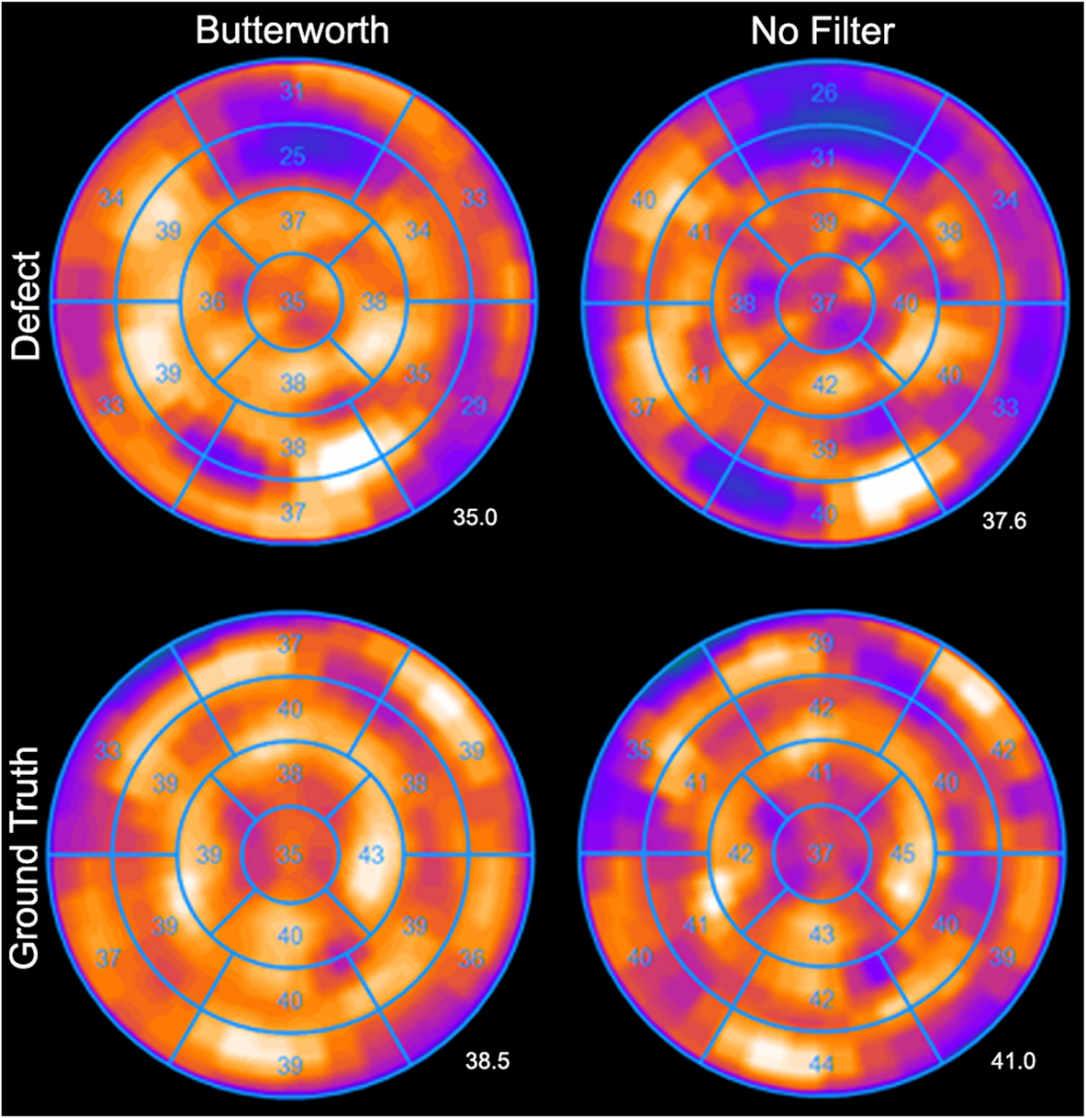
Bull’s eye plots of Butterworth filtered data (left panel) and unfiltered data (right panel). The values are in kBq/mL and depict one acquisition of the S-configuration with the defect in the mid-anterior segment (top) and ground truth (bottom). The number right below the Bull’s eye plot concerns the average global tracer uptake in kBq/mL

There was significant difference (*p* < 0.05) between the filtered and unfiltered uptake per segment for both the GT- and small SS-configuration (Fig. 4). However, the filtered data showed more homogenous uptake in the myocardium. The unfiltered data showed a more heterogeneous pattern and showed more areas of less uptake that do not contain defects, and therefore, the filtering was applied to the images in the remainder of this study. For the S-configuration, the smallest uptake (kBq/mL) was observed in the mid anterior segment, which contained the defect. Mean activity concentration per segment for this configuration with and without post-filtering is depicted in Supplemental Fig. S1.

### Patient cases

As a result of the phantom study, 7 iterations (and 10 subsets) and Butterworth post-filtering was applied for the image reconstruction of ten patient cases recruited for BMC injection between February 2017 and May 2019. Patient characteristics are summarized in Table 2. The average global tracer uptake in the myocardium (in kBq/mL), depicted in Table 2, was within the same range as the average global tracer uptake applied to the myocardium in the phantom study (Fig. 4; range 14.9–43.1 and 35.0–38.5, respectively). Furthermore, the minimum segmental tracer uptake was in the same range as the tracer uptake applied to the defect in the phantom study (Table 2; range 6.5–33.5 kBq/mL and Fig. 4; 25 kBq/mL, respectively). The median time between the SPECT/CT scans before and after BMC injection was 9 months. Association between the reversibility Bull’s eye plots in SUV_LBM_ before and after BMC injection is provided in Table 3. Eight patients showed significant difference (*p* < 0.05) in SUV_LBM_ between before and after the injection with a maximal improvement of 2.22 g/mL (patient no.7).

**Table 2:** Patient characteristics (n = 10)

Characteristic	Value
Gender	
Male	9
Female	1
Age (years)	69 (53–82)
Weight (kg)	82 (46–124)
Height (cm)	176 (160–187)
Time delay between BMC injection and imaging (mo.)	3 (1–13)
Activity received for MPS	
~ 500 MBq	8
~ 750 MBq	2
Average global tracer uptake (kBq/mL)	
Before BMC injection rest	25.7 (14.9–33.1)
Before BMC injection stress	27.3 (19.3–37.5)
After BMC injection rest	25.5 (18.6–43.1)
After BMC injection stress	30.1 (19.5–41.2)
Minimum segmental tracer uptake (kBq/mL)	
Before BMC injection rest	20.4 (7.2–27.8)
Before BMC injection stress	17.8 (6.5–29.1)
After BMC injection rest	19.3 (8.1–33.5)
After BMC injection stress	21.0 (8.4–27.8)
Medical history	
CABG	10
PCI	6
MI	4
ICD	1
BMC injection	2
PM	0
Medication	
Diuretics	3
Beta-blockers	10
Scanned bed position	
Prone position	7
Supine position	3
Ejection fraction (%)	
Rest before BMC	55 (24– > 60)
Stress before BMC	52 (21– > 60)
Rest after BMC	55 (25– > 60)
Stress after BMC	52 (21– > 60)

Table shows median and range. *BMC* bone marrow cell, *SPECT/CT* single photon emission computed tomography/computed tomography, *MPS* myocardial perfusion scintigraphy, *CABG* coronary artery bypass grafting, *PCI* percutaneous coronary intervention, *MI* myocardial infarction, *ICD* implantable cardioverter defibrillator, *PM* pacemaker

**Table 3:** Association between the relative mean difference (range), derived from reversibility Bull’s eye plots, in SUV_LBM_ (g/mL) before and after BMC injection

Patient no.	SUV_*LBM*_ (g/mL)		
	Pre treatment	Post treatment	*p*-value
1	0.60 (0.10 to 1.25)	0.19 (− 0.26 to 0.64)	**0.001**
2	− 0.55 (− 1.30 to 0.19)	0.19 (− 0.89 to 1.10)	**0.001**
3	− 0.74 (− 1.52 to 0.12)	0.32 (− 0.70 to 0.91)	**< 0.001**
4	0.75 (0.04 to 1.59)	− 0.42 (− 1.07 to 0.23)	**< 0.001**
5	− 1.11 (− 1.62 to − 0.67)	− 0.03 (− 0.68 to 0.45)	**< 0.001**
6	0.48 (− 0.09 to 1.16)	0.17 (− 0.55 to 0.98)	0.093
7	− 0.55 (− 1.13 to 0.14)	1.67 (0.62 to 2.39)	**< 0.001**
8	− 0.44 (− 1.03 to 0.08)	− 0.38 (− 0.67 to 0.14)	0.586
9	− 1.13 (− 1.69 to − 0.66)	− 0.30 (− 0.82 to 0.38)	**< 0.001**
10	2.06 (0.98 to 2.53)	− 0.54 (− 1.63 to 0.68)	**< 0.001**

## Discussion

To the best of our knowledge, this is the first study that evaluated Evolution for cardiac applications. The experimental part of this study evaluated the accuracy and precision of Evolution by utilizing various phantom experiments. To illustrate the feasibility of clinical application, ten patients, before and after BMC treatment, were included retrospectively. Seven iterations (10 subsets) and Butterworth post-filtering (cut off frequency 0.52 in cycles/cm, order of 5) were considered optimal for reconstruction based on convergence and noise level. Applying these settings, the average repeatability deviation (or precision) of all acquisitions was 2.91%. Moreover, the accuracy of Evolution using larger defects resulted in higher RC values (ranging from 0.64 to 0.75) compared to smaller defects (RC ranging from 0.52 to 0.74). Bull’s eye plots were generated to evaluate the uptake per segment.

RC values determined for both myocardium and defect were in general underestimated (i.e. < 1), except for the SS-configuration (Table 1 and Fig. 3). This underestimation is likely a result of partial volume effects. The spatial resolution of the system with a LEHR collimator is 7.4 mm full-width-at-half-maximum (FWHM) [16]. The resolution-modelling in Evolution has a positive effect on resolution, which resulted in an increment from 7.4 mm FWHM to 5.1–6.4 mm FWHM [16]. However, our results might differ since the resolution-modelling in Evolution is based on bone applications performed with LEHR collimators in H-mode and hence this provides only an indication of the improved spatial resolution for cardiac applications. Hypothetically, smaller defects are more affected by the spill-in effect of neighbouring tissues, like the myocardium, resulting in higher recovery. However, the thickness of the myocardial wall (~ 10 mm), producing the spill out effect, might explain the lower RC values. Similar results were reported in a cardiac phantom study [17] and a bone phantom study using the same SPECT/CT camera [8]. In addition, modelling the collimator detector response might also clarify the decrease in quantitative accuracy, since this is acknowledged to introduce blurring in the final reconstruction of SPECT data [18]. Furthermore, the findings showed higher accuracy in larger defects (11.8 mL) compared to smaller defects (2.6 mL), possibly explained by these partial volume effects. However, both defect size and SUV_LBM_ uptake (relative and absolute) are of clinical interest for accurately assessing response to treatment in patients with CAD. Therefore, the investigation of the spill-in and spill-out due to partial volume effects remains of interest for future cardiac SPECT studies.

The observed inhomogeneity (artefacts) in the Bull’s Eye plot might lead to difficulties in interpretation and may affect the quantitative treatment evaluation of MPS. Butterworth filtered data demonstrated a more homogeneous uptake compared to unfiltered data (Fig. 4), despite the comparable noise levels (Fig. 2c), and is therefore preferred. However, the recent quantification with Evolution was developed for the quantification of “hot spots” in mainly bone, lung and liver applications, while uniformity and contrast are more important to quantify defects (cold spots) for cardiac applications. A previously designed Evolution for (non-quantitative) cardiac applications combines the maximum a posteriori (MAP) algorithm [19] with the one-step-late (OSL) algorithm using a Green prior and median root prior (last iteration only) [20] to suppress formation of hot spots due to noise in the acquired projections. Therefore, it would be worthwhile to investigate a new variant of Evolution that combines quantification (compensation for collimator–detector response, attenuation and scatter correction, and resolution recovery) with MAP and OSL for quantitative cardiac applications.

This study contains some limitations. First, no dedicated dynamic cardiac phantom was used in this experimental study. Therefore, the effects of cardiac motion, a challenge among cardiac scanning, causing artefacts and distortions in image datasets, could not be investigated [21]. Second, only Evolution was used for quantitative SPECT; hence, no performance comparison could be made with other quantification software packages such as SUV SPECT (Hermes Medical Solutions, Stockholm, Sweden). Third, the reported results are specific to one vendor and one implementation of iterative reconstruction.

Finally, the goal of the patient cases was to illustrate the clinical applicability of quantitative SPECT in the current MPS clinical workflow for response monitoring. This feasibility study showed that it was possible to apply the phantom settings to clinical patient data and generate Bull’s eye plots in SUV_LBM_. Significant differences in SUV_LBM_ on the scan prior to and after BMC injection were found in eight patients, indicating that BMC injection has had an effect that led to a change in tracer uptake. The other two patients showed no significant difference. This implies that no change in uptake occurred after BMC injection. However, larger sized prospective clinical trials should be executed for validation and implementation of this technique and in order to investigate the accuracy, clinical applicability and clinical value of quantitative SPECT for therapy response monitoring in patients with CAD.

## Conclusion

Quantification of ^99m^Tc related SPECT/CT for response monitoring in patients with CAD seems feasible when 7 iterations (10 subsets), Butterworth post-filtering and manual delineation on CT images are used. To show sufficient evidence for the use of Evolution in clinical practice for treatment response monitoring, validation should take place in a future prospective clinical trial, studying a large patient cohort. Quantitative SPECT, however, is promising and might extend the diagnostic potential of standard MPI.

## Supplementary Information

**Additional file 1.**

## Data Availability

The datasets generated during and/or analysed during the current study are available from the corresponding author on reasonable request.
