# Supplementary material for: Experimental validation of absolute SPECT/CT quantification for response monitoring in patients with coronary artery disease
Source: EJNMMI Phys. 2021 Jun 16;8:48. doi: 10.1186/s40658-021-00393-4 (PMC8208344; doi:10.1186/s40658-021-00393-4)
Supplement: Supplementary file 1 — Additional file 1. [file 40658_2021_393_MOESM1_ESM.docx]

**Experimental validation of absolute SPECT/CT quantification for response monitoring in patients with coronary artery disease**

EJNMMI Physics

Alina van de Burgt, MSc, Petra Dibbets-Schneider, BSc, Cornelis H. Slump, PhD, Arthur J.H.A. Scholte, MD PhD, Douwe E. Atsma, MD PhD, Lioe-Fee de Geus-Oei, MD PhD and Floris H.P. van Velden, PhD

First Author: Alina van de Burgt, MSc.

l.oei@utwente.nl


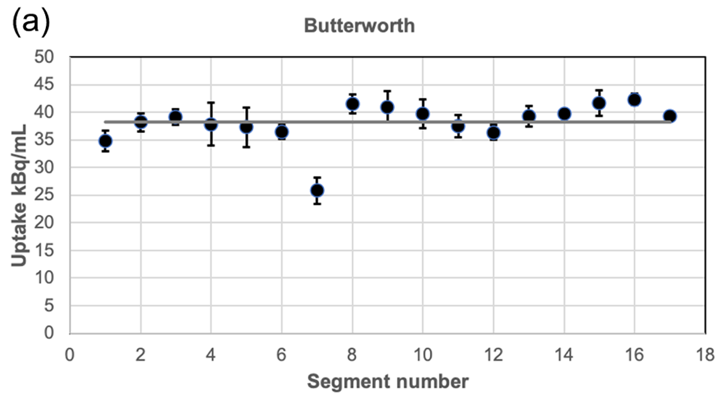

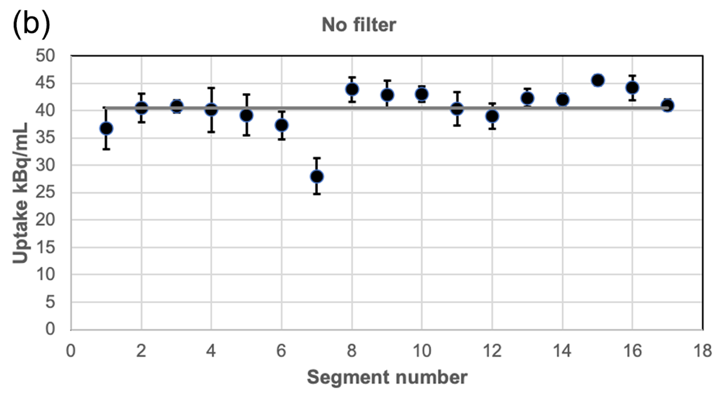


**Fig. S1** Mean activity concentration for the defect per segment for the S-configuration, obtained with (**a**) and without (**b**) post-filtering. The grey line represents the mean of the 17 segments. The error bars indicate the standard deviation per segment obtained over 6 acquisitions.
